# THE RELATIONSHIP BETWEEN ROLE CAPTIVITY, COPING STRATEGIE,S AND DEMENTIA FAMILY CAREGIVER MENTAL HEALTH

**DOI:** 10.1093/geroni/igad104.2228

**Published:** 2023-12-21

**Authors:** Abigail Poe, Loreli Alvarez, Mallory Wood, Frank Puga

**Affiliations:** University of Alabama at Birmingham, Birmingham, Alabama, United States; The University of Alabama at Birmingham, Birmingham, Alabama, United States; University of Alabama at Birmingham, Birmingham, Alabama, United States; University of Alabama at Birmingham, Birmingham, Alabama, United States

## Abstract

Informal caregivers of individuals living with dementia have an increased risk for experiencing depressive and anxiety symptoms. The stress-process model posits that situational stressors and stress moderators, such as role captivity and coping strategies, predict worse mental health outcomes; however, relatively little is known about the relationship between role captivity and specific coping strategies dementia caregivers use to manage caregiving-related stress. The purpose of this study was to examine relationships between role captivity, coping strategies, and depressive and anxiety symptoms. A preliminary analysis was conducted on a sample of community-dwelling family caregivers (N=30) from an ongoing national study on daily caregiving experiences. Data were analyzed using regression analysis. Dementia family caregivers reported moderate anxiety symptoms, measured by the GAD-7 (M= 11.86, SD= 4.39) and moderate depressive symptoms, measured by the CESD, (M=19.59, SD= 12.67). The sample’s mean age was 54.7 years old and 76.7% female. Role captivity was positively associated with depression (β=2.98, t(28) = 3.94, p< 0.001) and anxiety (β= 0.92, t(28) = 3.31, p< 0.01) among dementia caregivers. Avoidant coping was positively associated role captivity (β=0.31, t(28) = 4.27, p< 0.001). The results of this study suggest role captivity is associated with an increase in depression and anxiety among dementia caregivers. Further, avoidant coping is associated with an increase in role captivity reported by caregivers. The findings from this study support the benefit of interventions that help dementia family caregivers in their role and promote the use of positive coping strategies.

